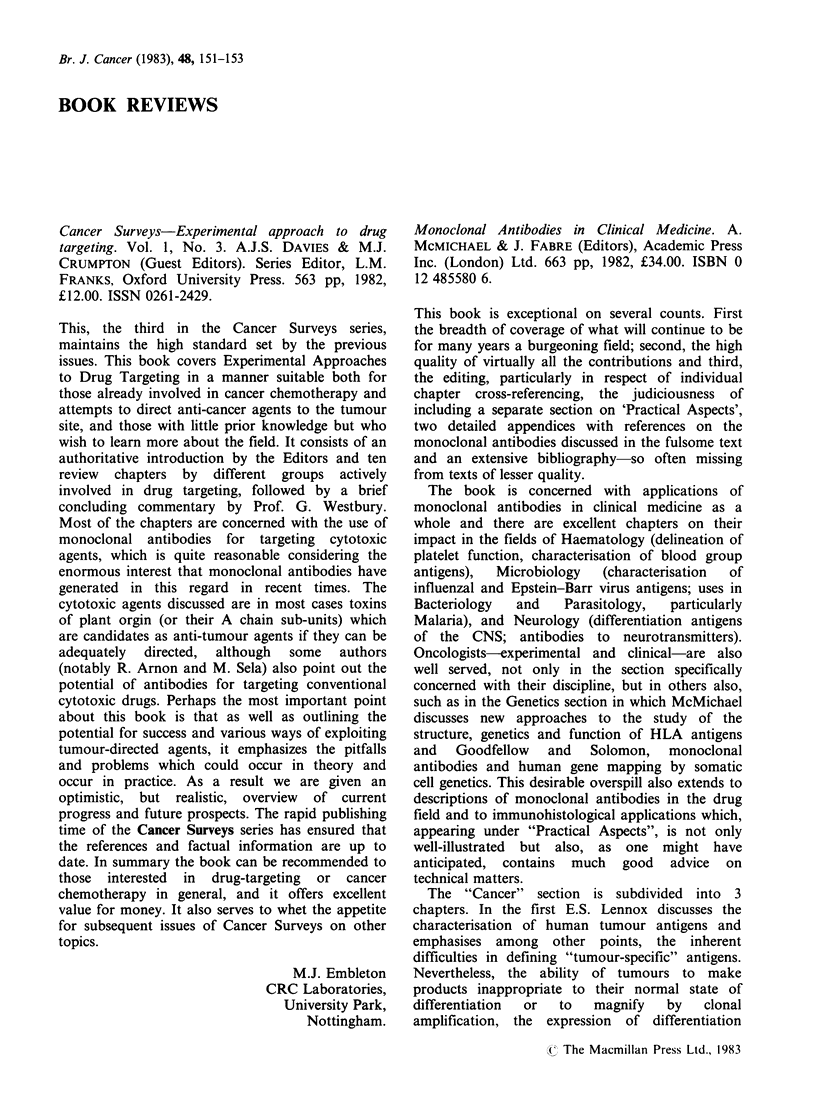# Cancer Surveys—Experimental approach to drug targeting

**Published:** 1983-07

**Authors:** M.J. Embleton


*Br. J. Cancer* (1983), **48**, 151–153

### BOOK REVIEWS

*Cancer Surveys—Experimental approach to drug targeting*. Vol. 1, No. 3. A.J.S. DAVIES & M.J. CRUMPTON (Guest Editors). Series Editor, L.M. FRANKS, Oxford University Press. 563 pp, 1982, £12.00. ISSN 0261-2429.

This, the third in the Cancer Surveys series, maintains the high standard set by the previous issues. This book covers Experimental Approaches to Drug Targeting in a manner suitable both for those already involved in cancer chemotherapy and attempts to direct anti-cancer agents to the tumour site, and those with little prior knowledge but who wish to learn more about the field. It consists of an authoritative introduction by the Editors and ten review chapters by different groups actively involved in drug targeting, followed by a brief concluding commentary by Prof. G. Westbury. Most of the chapters are concerned with the use of monoclonal antibodies for targeting cytotoxic agents, which is quite reasonable considering the enormous interest that monoclonal antibodies have generated in this regard in recent times. The cytotoxic agents discussed are in most cases toxins of plant orgin (or their A chain sub-units) which are candidates as anti-tumour agents if they can be adequately directed, although some authors (notably R. Arnon and M. Sela) also point out the potential of antibodies for targeting conventional cytotoxic drugs. Perhaps the most important point about this book is that as well as outlining the potential for success and various ways of exploiting tumour-directed agents, it emphasizes the pitfalls and problems which could occur in theory and occur in practice. As a result we are given an optimistic, but realistic, overview of current progress and future prospects. The rapid publishing time of the **Cancer Surveys** series has ensured that the references and factual information are up to date. In summary the book can be recommended to those interested in drug-targeting or cancer chemotherapy in general, and it offers excellent value for money. It also serves to whet the appetite for subsequent issues of Cancer Surveys on other topics.

M.J. Embleton
CRC Laboratories,
University Park,
Nottingham.